# MYC-dependent downregulation of telomerase by FLT3 inhibitors is required for their therapeutic efficacy on acute myeloid leukemia

**DOI:** 10.1007/s00277-017-3158-8

**Published:** 2017-10-27

**Authors:** Xiaolu Zhang, Bingnan Li, Jingya Yu, Jenny Dahlström, Anh Nhi Tran, Magnus Björkhom, Dawei Xu

**Affiliations:** 1 Center for Hematology, Department of Medicine and Center for Molecular Medicine, Karolinska University Hospital Solna and Karolinska Institutet, SE-171 76 Stockholm, Sweden; 2 Department of Clinical Genetics, Karolinska University Hospital and Karolinska Institutet, SE-171 76 Stockholm, Sweden

**Keywords:** AML, FLT3ITD, hTERT, Targeted therapy, Telomerase

## Abstract

The somatic mutation of FLT3 occurs in 30% of acute myeloid leukemia (AML), with the majority of mutations exhibiting internal tandem duplication (ITD). On the other hand, the induction of telomerase reverse transcriptase (hTERT) and the activation of telomerase is a key step in AML development. Here, we sought to determine whether FLT3ITD regulates hTERT expression in AML cells and whether hTERT expression affects FLT3 inhibitors’ therapeutic efficacy on AML. FLT3ITD-harboring AML cell lines and primary cells treated with the FLT3 inhibitor PKC412 displayed a rapid decline in the levels of hTERT mRNA and telomerase activity. Moreover, PKC412 inhibited *hTERT* gene transcription in a c-MYC-dependent manner. The ectopic expression of hTERT significantly attenuated the apoptotic effect of PKC412 on AML cells. Mechanistically, hTERT enhanced the activity of FLT3 downstream effectors or alternative RTK signaling, thereby enhancing AKT phosphorylation, in AML cells treated with PKC412. Collectively, PKC412 downregulates hTERT expression and telomerase activity in a MYC-dependent manner and this effect is required for its optimal anti-AML efficacy, while hTERT over-expression confers AML cells resistance to a targeted therapeutic agent PKC412. These findings suggest that the functional interplay between FLT3ITD and hTERT contributes to the AML pathogenesis and interferes with the efficacy of FLT3ITD-targeted therapy.

## Introduction

Acute myeloid leukemia (AML) is a heterogeneous group of hematological malignancies, characterized by a differentiation block and aberrant clonal growth of hematopoietic blasts, and results from specific recurrent cytogenetic abnormalities and somatic mutations. FMS-like tyrosine kinase 3 (FLT3), a type III receptor tyrosine kinase (RTK), is one of the most frequently mutated genes and occurs in approximately one third of AML, with the majority of mutations exhibiting the internal tandem duplication (ITD) in its justamembrance domain [1, 2]. FLT3ITD is believed as a leukemogenesis driver and predictor for poor outcome in AML patients [2–6]. In the last years, a panel of small-molecule tyrosine kinase inhibitors (TKIs) targeting FLT3 have been developed and tested for their clinical efficacy [5]. Despite the observed clinical responses and the straightforward therapeutic mechanism, it remains incompletely understood whether FLT3LTD has other biological activities. In addition, many AML patients who receive FLT3TKIs eventually acquire drug resistance [5, 7–9]. It has been well-characterized that the occurrence of new mutations in the FLT3 genome is an important mechanism contributing to patients’ resistance to FLT3TKIs [5, 7–9]. However, such new mutations were only found in a fraction of patients who have developed FLT3TKI resistance, and there likely exist other elements affecting the therapeutic efficacy of FLT3TKIs [9–12]. Taken together, better understanding of the biological functions of FLT3ITD and of the mechanisms underlying FLT3TKIs is certainly required to fully elucidate the role of FLT3 activation in leukemogenesis and to improve the therapeutic efficacy of FLT3TKIs.

Telomerase is an RNA-dependent DNA polymerase responsible for synthesizing telomeric DNA at chromosome ends [13, 14]. The core telomerase complex consists of two components: the rate-limiting, catalytic subunit telomerase reverse transcriptase (hTERT) and the ubiquitously expressed RNA template hTERC [13] [14, 15]. Most normal human cells lack telomerase activity due to the stringent transcriptional repression of the *hTERT* gene, while the induction of hTERT expression and telomerase activation is in general a prerequisite step for malignant transformation of human cells [13, 15]. Evidence has also accumulated that hTERT possesses many other biological activities in addition to its canonical telomere-lengthening function [13]. For instance, hTERT was shown to facilitate cancer progression by inducing epithelial-to-mesenchymal transition and a cancer stem cell phenotype [16]. In addition, hTERT protects cancer cells from apoptosis induced by chemotherapeutic drugs and other insults [17–23]. It is thus evident that hTERT or telomerase plays multiple roles in cancer development, progression, and treatment.

Like the majority of human malignancies, AML displays widespread telomerase activation and hTERT expression [24]. However, a number of important issues have not so far been explored yet: (i) whether FLT3ITD regulates hTERT expression or telomerase activity in AML cells and (ii) hTERT or telomerase was shown to attenuate chemotherapeutic and other drug-induced apoptosis [17–20, 22, 25], but it is unclear whether hTERT interferes with the efficacy of FLTTKI-targeted therapy. In the present study, we address these issues by dissecting the regulatory and functional interplay between FLTITD and hTERT in AML.

## Materials and methods

### Cell lines, culture conditions, and PKC412 treatment

FLT3ITD-harboring AML cell lines MV4, 11 and MOLM-13, acute promyelocytic leukemia cell line HL60, and cervical cancer cell line HeLa were used in the present study and cultured at 37 °C/95% air/5% CO_2_ in RPMI 1640 medium (Life Technologies, Paisley, Scotland, UK) containing 10% fetal calf serum, 100 units/ml penicillin, and 2 mM l-glutamine. The specific FLT3 inhibitor PKC412 (Sigma-Aldrich, Buchs, Switzerland) [26] was diluted in DMSO, and cells were incubated with different concentrations of PKC412 for various time periods.

### Primary AML cell isolation and culture

Primary FLT3ITD-carrying AML cells were derived from two AML patients. Patient 1: 22 years old, diagnosed as acute promyelocytic leukemia-carrying t(15;17) and FLT3ITD, WBCC = 0.5 × 10^9^/l, dominance of promyelocytes and blasts 0%. The treatment included all-trans retinoic acid (ATRA) and idarubicin/cytosine-arabinoside as induction, two consolidation courses with the same agents, followed by ATRA every 3 months for 2 years. The patient was in molecular CR. Patient 2: 79 years old, diagnosed as AML with del(20) and FLT3ITD, WBCC = 161.8 × 10^9^/l with blasts 91.5%. The patient died prior to treatment. Patient peripheral blood was drawn, and AML cells were isolated by Lymphoprep gradient centrifugation (Nycomed, Oslo, Norway). Isolated AML cells were subsequently incubated in complete medium in the absence or presence of PKC412 as described above. The study was approved by the Stockholm Regional Ethics Review Committee, and written informed consent was obtained from the subjects. All experiments were performed in accordance with relevant guidelines and regulations.

### RNA extraction, reverse transcription, and quantitative PCR

Total cellular RNA was extracted using the Trizol kit (Life Technology) according to the manufacturer’s protocols. Complementary DNA (cDNA) was synthesized using random primers (N6) (Amersham, Buckinghamshire, UK) and M-MLV reverse transcriptase. The PCR primers are listed in Table 1. β2-Microglobulin (β2-M) expression was used as a control for RNA loading and RT efficiency and amplified in parallel. qPCR was carried out in an ABI7700 sequence detector (Applied Biosystems, Foster City, CA) using a SYBR Green kit (Applied Biosystems) with triplicates. Levels of hTERT, c-MYC, c-KIT, DOK3, and SULF2 messenger RNA (mRNA) were calculated based on the threshold cycle (CT) values and normalization of human β2-M expression.

**Table 1:** PCR primers used in the present study

hTERT
5′-CGGAAGAGTGTCTGGAGCAA-3′ (forward)
5′-GGATGAAGCGGAGTCTGGA-3′ (reverse)
c-MYC
5′-TACCCTCTCAACGACAGCAGCTCGCCCAACTCCT-3′ (forward)
5′-TCTTGACATTCTCCTCGGTGTCCGAGGACCT-3′ (reverse)
c-KIT
5′-TCATGGTCGGATCACAAAGA-3′ (Forward)
5′-AGGGGCTGCTTCCTAAAGAG-3′ (Reverse)
DOK3
5′-GTCCCCATGGAGGAAAACTC-3′ (Forward)
5′-AAGTGGTAGGGCCAGCTGTA-3′ (Reverse)
SULF2
5′-CCGCCCAGCCCCGAAACC-3′ (Forward)
5′-CTCCCGCAACAGCCACACCTT-3′ (Reverse)

### Assessment of telomerase activity

Telomerase activity was determined using a commercial telomerase PCR ELISA kit (Roche Diagnostics Scandinavia AB, Stockholm, Sweden) according to the manufacturer’s instruction. Total cellular proteins were extracted using CHAPS lysis buffer. For each assay, 0.5 μg of protein was used, and 26 PCR cycles were performed after the telomerase-primer elongation reaction. The PCR products were detected with an ELISA color reaction. The measured telomerase activity was expressed as absorbance [optimal density (OD) in arbitrary units].

### Western blot

Total cellular proteins were extracted using RIPA lysis buffer. Twenty micrograms of protein was subjected to sodium dodecyl sulfate-polyacrylamide gel electrophoresis and transferred to a PVDF membrane. The membranes were probed with the specific antibody against FLT3, p-FLT3, Akt, and p-Akt (Cell Signaling Technology, Boston, USA) or c-MYC (Santa Cruz Biotechnologies) followed by anti-mouse or rabbit horseradish peroxidase-conjugated IgG and developed with the enhanced chemiluminescent method (SuperSignal West Pico Chemiluminescent Substrate, Thermo Scientific). β-Actin immunoblotting was performed in parallel as a loading control.

### hTERT promoter activity assay

The hTERT promoter reporter plasmid p181^wt^ harboring the core promoter sequence of the hTERT 5′-flanking region and its mutant variant (p181^MYC−^) lacking the functional c-MYC motifs (E-boxes) were described previously [27, 28]. MV4,11 and MOLM-13 cells cultured in 24-well plates at 0.5 × 10^6^ were transfected with p181^wt^ and p181^MYC−^ plasmids using Lipofectamine2000 (Life Technology) according to the manufacturer’s protocol, followed by the treatment with PKC412. Luciferase activity in the cell lysates was determined by using a dual-luciferase reporter assay system (Promega, WI) 24 h post-transfection of the promoter reporter.

### Puro. Cre-hTERT promoter-driven GFP plasmid and Lenti-III-HA-GFP-hTERT vectors

The h3.4k-GFP plasmid containing 3.4-kb hTERT promoter (+ 1 to − 3405, ATG as + 1) was obtained from Dr. Pei-Rong Huang (National Taiwan University) [29], and the 3.4-kb hTERT promoter fragment was inserted into a pPuro. Cre empty vector (Addgene) just upstream of the *GFP* gene. For pLenti-III-HA-GFP-hTERT vector construction, plenti-III-HA empty vector was bought from Applied Biological Materials Inc. (BC, Canadia), a 4.5-kb GFP-hTERT fragment was cut from pBabe-hygro-GFP-hTERT (addgene) and inserted into pLenti-III-HA. A control plasmid (pLenti-BMN-GFP) was a gift from Rudbeck Laboratory, Uppsala University. The vectors were then packaged, and viral particles were collected to infect AML cells to make hTERT promoter-driven GFP cells and hTERT-over-expressed cells.

### Cell cycle and apoptosis analyses

Stable MOLM-13 cells infected with control pBMN or hTERT-expressing lentiviral vectors were treated with 0.1 μM PKC412 and harvested at 24 h, fixed with 70% ethanol at + 4 °C overnight, and stained with RNAse A (0.5 μg)-containing propidium iodide (PI, 50 μg/ml). Cell cycle distribution and apoptotic cells were determined using flow cytometry with ModFit.

### cDNA array

MOLM-13 cells infected with control pBMN or hTERT-expressing lentiviral vectors were treated with 0.1 μM PKC412 for 12 h, and total RNA was extracted. The affymetrix Human Gene 1.0 ST Array was performed. The fold change in gene expression between DMSO- and PKC412-treated cells was then calculated.

### Statistical analyses

The comparison of mRNA expression, promoter activity, telomerase activity, viability, and cell cycle analysis between control and experimental groups was made using Student’s *t* test or one-way ANOVA followed by LSD test. All the tests were two-tailed and computed using SPSS software. *P* values less than 0.05 were considered statistically significant.

## Results

### Downregulation of hTERT expression by the FLT3TKI PKC412 in AML cells (MV4,11 and MOLM-13) carrying FLT3ITD mutation

**Fig. 1:**
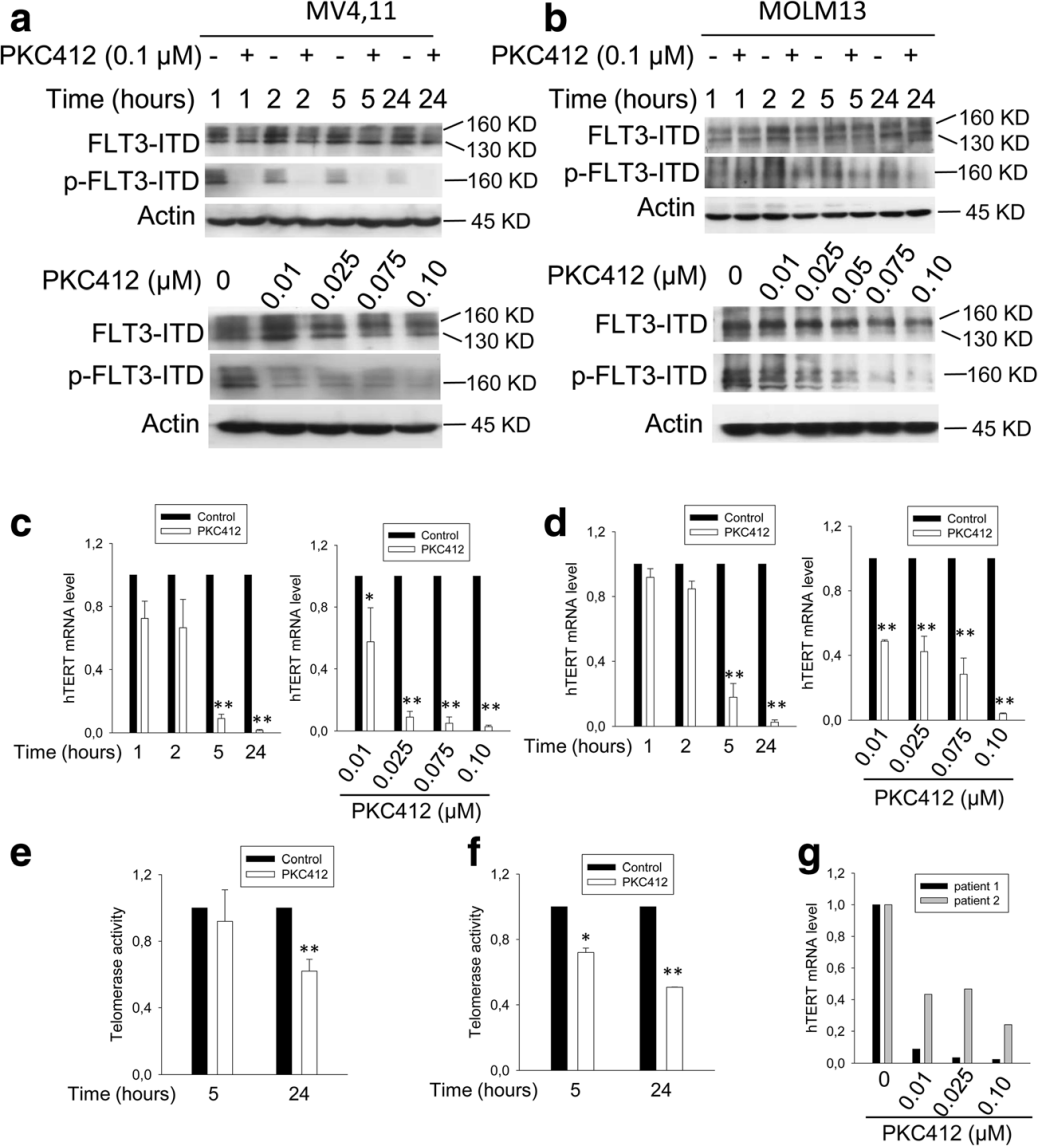
PKC412, a specific FLT3ITD inhibitor, downregulates hTERT expression and telomerase activity in FLT3ITD-carrying AML cells. **a**, **b** The time- and dose-dependent inhibitions of FLT3 phosphorylation in PKC412-treated MV4,11 (**a**) and MOLM-13 cells (**b**). Immunoblotting was performed. and one of three independent experiments is shown. **c**, **d** PKC412-induced downregulation of hTERT expression and telomerase activity in MV4,11 (**c**) and MOLM-13 cells (**d**) in time- and dose-dependent manners. hTERT mRNA expression was determined using qPCR, and the level of hTERT mRNA was arbitrarily calculated based on CT values normalized with β2-M expression. **e**, **f** The downregulated telomerase activity in MV4,11 (**e**) and MOLM-13 cells (**f**) treated with PKC-412. The level of telomerase activity was assessed using a telomerase ELISA kit and expressed as absorbance in arbitrary units. **g** The downregulation of hTERT mRNA in primary FLT3ITD-carrying leukemic cells treated with PKC-412. Leukemic cells were derived from two patients with AML and incubated in the presence of different concentrations of PKC412 for 24 h. hTERT mRNA expression level was determined using qPCR. *P < 0.05; ** *P* < 0.01. Student’s *t* test was performed. All the results shown were from three independent experiments

PKC412 is a TKI specifically targeting FLT3ITD, and as expected, the PKC412 treatment of MV4,11 and MOLM-13 cells inhibited FLT3 phosphorylation and activity (Fig. 1a, b). To determine the effect of PKC412 on hTERT expression, we incubated FLT3ITD+ cells with PKC412. The PCR analysis showed that MV4,11 cells displayed a time-dependent downregulation of hTERT mRNA expression in the presence of PKC412 at 0.1 μM (Fig. 1c). This inhibitory effect was also dose-dependent, and hTERT mRNA became undetectable in MV4,11 cells treated with PKC412 at 0.1 μM for 24 h (Fig. 1c). Almost identical results were obtained in PKC412-treated MOLM-13 cells, another FLT3ITD+ AML line (Fig. 1d). In addition, we treated wild-type FLT3-carrying HL60 cells and HeLa cells with PKC412 and did not observe detectable changes in hTERT mRNA levels in these cells (data not shown), which suggests that PKC412 inhibited hTERT expression via FLT3ITD. Consistent with diminished hTERT mRNA expression, telomerase activity was significantly repressed in MV4,11 and MOLM-13 cells treated with PKC412 (Fig. 1e, f). However, certain levels of residual activity remained by 24 h, likely due to the long half-life of telomerase [30, 31].

### hTERT inhibition in primary FLT3ITD-carrying AML cells treated with PKC412

Given the observation described above, we next sought to determine whether this is the case in primary AML cells. Primary FLT3ITD-positive leukemic cells derived from two newly diagnosed AML patients were incubated with PKC412, and hTERT transcripts were then assessed. As shown in Fig. 1g, PKC412 treatment of these AML cells similarly led to the substantial decline in hTERT mRNA levels.

### The inhibition of the hTERT promoter activity by PKC412

**Fig. 2:**
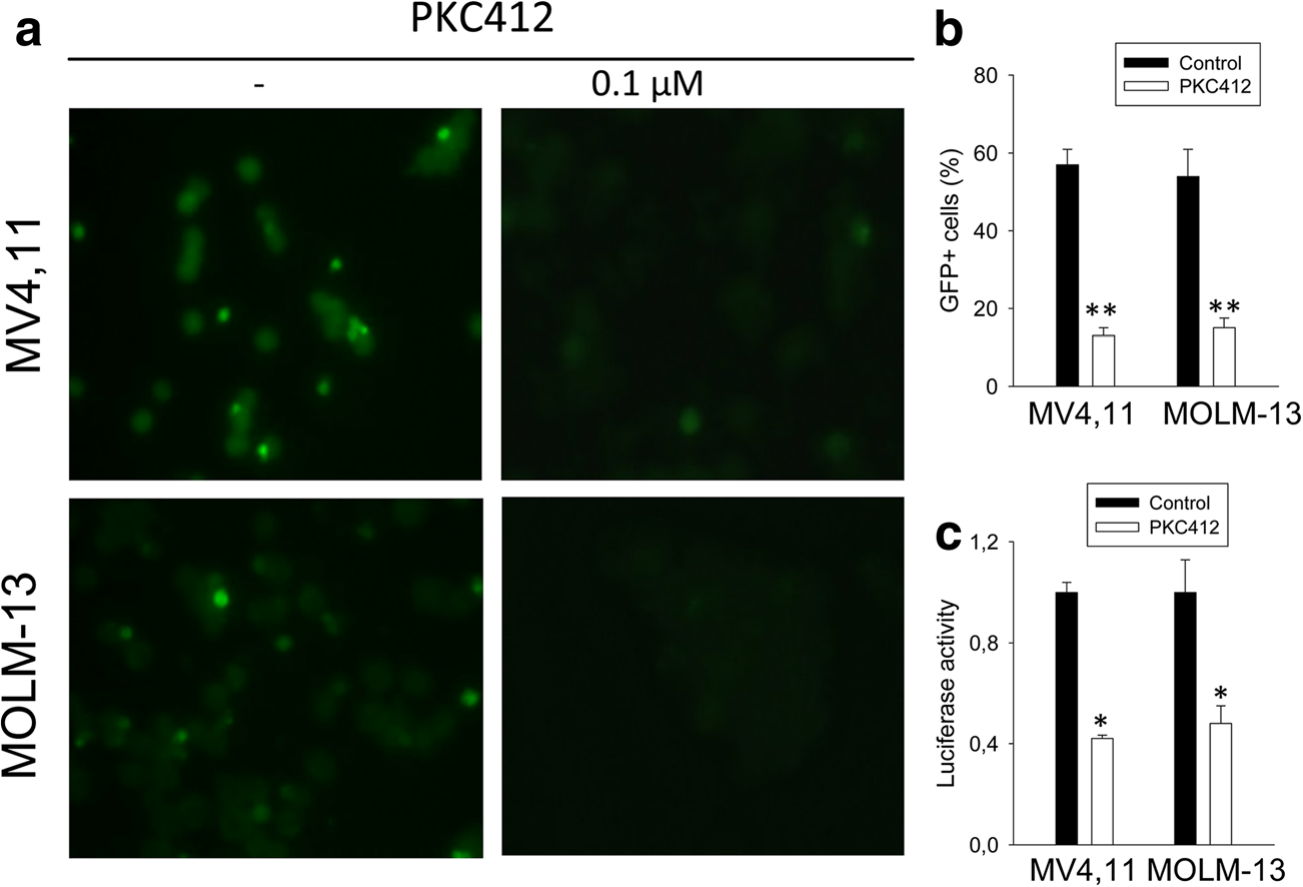
Repression of the hTERT promoter activity by PKC412. **a** A 3.4-kb hTERT promoter-driven GFP expression was inhibited by PKC412. MV4,11 and MOLM-13 cells were infected with a GFP expression vector driven by a 3.4-kb hTERT promoter and then incubated with or without PKC412. GFP-positive cells were examined and counted under fluorescence microscopy. Right panel: DMSO-treated control cells. Left panel: PKC412-treated cells. **b** GFP+ cells with and without PKC412 were counted as above and expressed as percentage of total cells. **c** PKC412-mediated inhibition of the wild-type hTERT promoter activity (p181^wt^). MV4,11 and MOLM-13 cells transfected with p181^wt^ plasmids were treated with PKC412 or DMSO, and luciferase activity was assessed after 24 h (see the “Materials and method” section for details). *P < 0.05; ** *P* < 0.01. Student’s *t* test was performed. All the results shown were from three independent experiments

We next determined the mechanism responsible for PKC412-mediated downregulation of hTERT expression in the FLT3ITD-carrying cells. To assess whether PKC412 regulated hTERT transcription, we infected MV4,11 and MOLM-13 cells with a GFP expression vector driven by a 3.4-kb-long hTERT promoter and then treated the cells with PKC412. As shown in Fig. 2a, b, there were about 50% of GFP-positive cells in the DMSO-containing culture while the presence of PKC412 led to diminished GFP+ cells. We further transfected the same cells with a core hTERT promoter reporter construct (p181) and then incubated them with DMSO or PKC412. The hTERT promoter activity, reflected as the level of luciferase activity, was significantly inhibited in the cells exposed to PKC412 compared to the DMSO-treated ones (Fig. 2c). Thus, PKC412 inhibited the hTERT transcription and clearly, the hTERT proximal promoter was sufficient for its inhibitory effect.

### The MYC-dependent inhibition of the hTERT transcription activity by PKC412

**Fig. 3:**
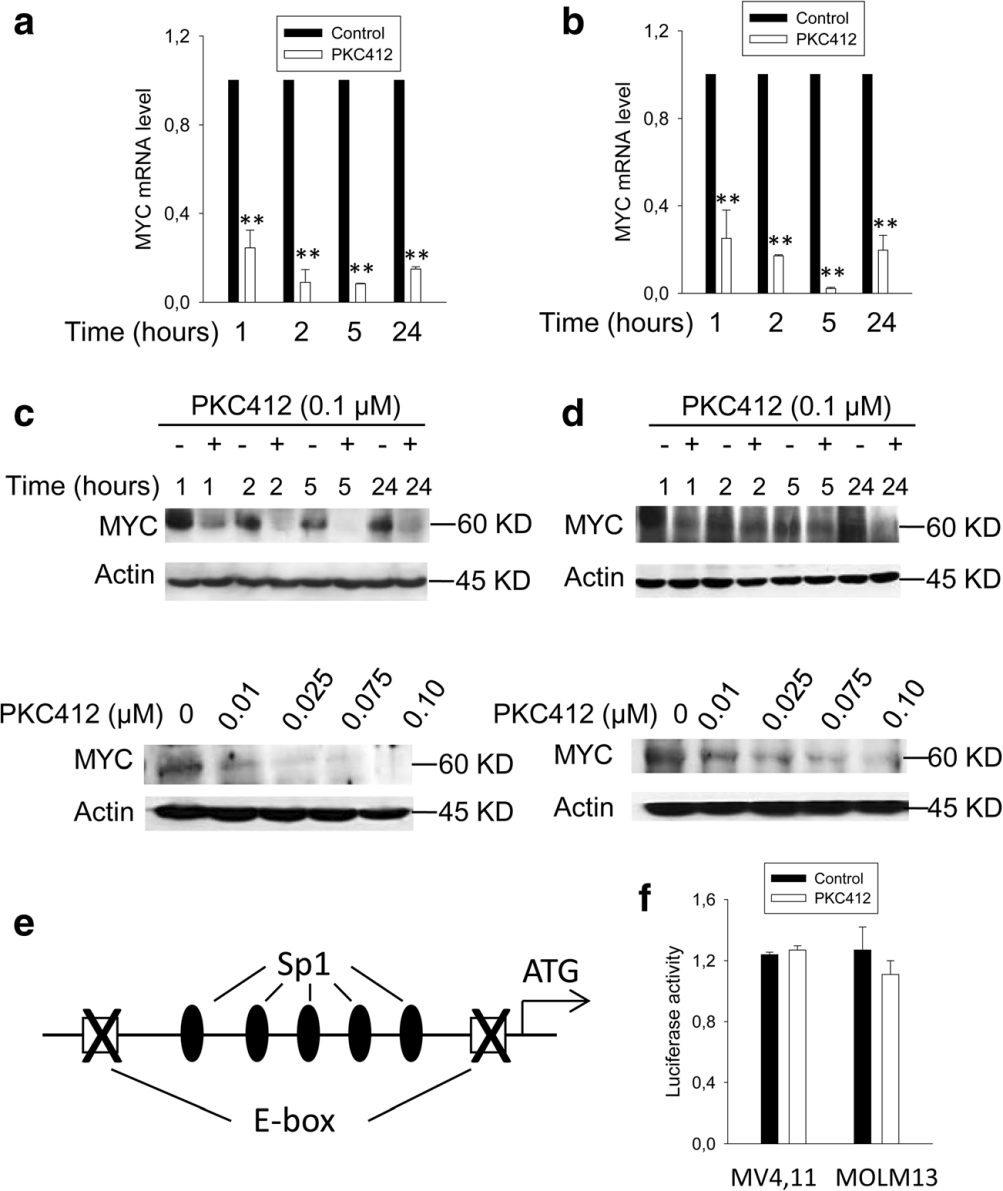
MYC-dependent repression of hTERT promoter activity by PKC412. **a**, **b** The diminished MYC mRNA in PKC412-treated MV4,11 (**a**) and MOLM-13 cells (**b**). The cells were incubated with PKC412 at 0.1 μM for different time periods and then analyzed for c-MYC mRNA using qPCR. **c**, **d** Time- and dose-dependent downregulations of MYC protein expression in PKC412-treated MV4,11 (**c**) and MOLM-13 cells (**d**). Immunoblotting was performed to determine MYC protein levels. **e**, **f** The abolishment of PKC412-mediated hTERT promoter repression by the disruption of E-boxes. **e** Schematic of the hTERT core promoter, showing the locations of two E-boxes relative to the translational start codon (ATG). **f** The hTERT core promoter-carrying vector was mutated to disrupt two E-boxes; transfected into MV4,11 and MOLM-13 cells; and then treated with PKC412 or DMSO for 24 h. Luciferase activity in the cell lysates was determined by using a dual-luciferase reporter assay system (Promega, WI). Of note, the results shown in both Fig. 2c and **f** were obtained from the same experiments and normalized to their controls (DMSO-treated). *P < 0.05; ** *P* < 0.01. Student’s *t* test was performed. All the results shown were from at least three independent experiments

It is well-established that c-MYC is the core transcription factor in transactivating the *hTERT* gene [27, 32], and we thus examined the link between FLT3ITD and c-MYC in regulating hTERT transcription. The treatment of FLT3ITD-carrying cells with PKC412 led to a fast and robust inhibition of c-MYC mRNA and protein expression in time- and dose-dependent manners, which preceded a decline in hTERT expression (Fig. 3a–d). To determine whether this downregulation of c-MYC expression is responsible for diminished hTERT expression in PKC412-treated cells, we transfected MV4,11 and MOLM13 cells with wild-type (wt) hTERT core promoter-harboring vectors (Fig. 2c) and its MYC binding site-deleted counterparts (Fig. 3e, f), respectively. Those cells were then exposed to PKC412. As shown above (Fig. 2c), wt hTERT promoter activity declined significantly in PKC412-treated cells compared to that in control cells (DMSO-treated). However, PKC412 did not affect the hTERT promoter activity any longer once two MYC binding sites on the promoter were disrupted (Fig. 3f). Taken together, PKC412-mediated repression of the hTERT transcription is MYC-dependent.

### The attenuation of PKC412-mediated AML cell apoptosis by the ectopic expression of hTERT

**Fig. 4:**
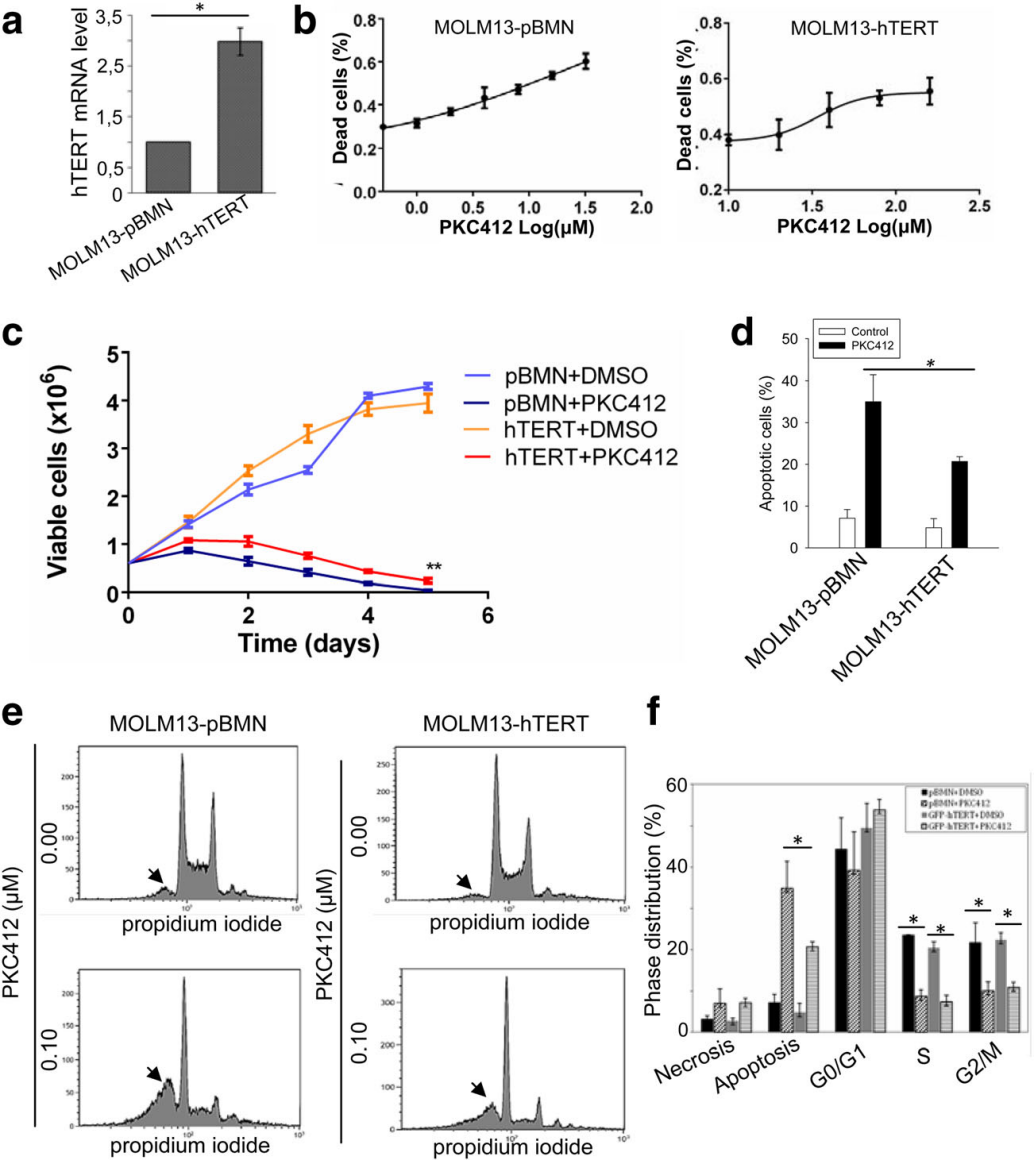
The attenuation of PKC412-induced apoptosis by ectopic hTERT expression in MOLM-13 cells. **a** MOLM-13 cells were infected with either control empty vector (pBMN) or hTERT-expressing vector to generate two sublines: MOLM-13-pBMN and MOLM-13-hTERT. The over-expression of hTERT in MOLM-13-hTERT cells was demonstrated using qPCR assay. **b** Cells were incubated with different concentrations of PKC412 for 48 h and IC_50_ then determined. Left panel: MOLM-13-pBMN cells and IC_50_ 17.2 μM. Right panel: MOLM-13-hTERT cells, IC_50_ 34.1 μM. **c** Cells were treated with 0.0125 and 0.1 μM PKC412 for 120 h, and viable cells were counted every 24 h. **P < 0.01. **d**, **e** hTERT-mediated attenuation of PKC412-induced apoptosis. MOLM-13-pBMN and MOLM-13-hTERT were treated with PKC412 at 0.1 μM and then analyzed for apoptotic cells using FACS. Representative FCCS graphs were shown in **e**, and the arrowheads point to the sub-G1 fractions (apoptotic cell population). *P < 0.05; ** *P* < 0.01. One-way ANOVA followed by LSD test was performed. **f** Cell cycle distributions in PKC412-treated cells. All the results shown were from three independent experiments

Having demonstrated the downregulation of hTERT expression by PKC412 treatment in FLT3ITD-harboring AML cells, we further asked whether this PKC412 effect was associated with its AML cell killing. For this purpose, we made a variant subline of MOLM-13 cells that ectopically expressed hTERT (MOLM-13-hTERT) (Fig. 4a). The control counterparts were infected with the empty vectors pBMN (MOLM-13-pBMN). These two sublines were assessed for their IC_50_ by incubating them with different concentrations of PKC412 for 48 h. As shown in Fig. 4b, IC_50_ was 17.2 and 34.1 μM for MOLM-13-pBMN (left panel) and MOLM-13-hTERT (right panel), respectively. These cells were further incubated with a low concentration of PKC412 (0.0125 μM) for different time periods (up to 120 h). The PKC412 treatment led to decreased viable cell numbers of both sublines; however, by 120 h, almost all MOLM-13-pBMN cells were dead whereas a fraction of MOLM-13-hTERT cells remained alive (*P* = 0.009) (Fig. 4c). Consistent with cell counting results, the FACS analysis revealed that PKC412 induced apoptosis in 35% of these cells, and the ectopic hTERT expression significantly attenuated apoptosis of MOLM-13 cells mediated by PKC412 (18%) (MOLM-13-hTERT vs MOLM-13-pBMN cells, *P* = 0.02) (Fig. 4d, e). In addition, PKC412 significantly decreased cells at S and G2/M of both MOLM-13-hTERT and MOLM-13-pBMN cells (Fig. 4f).

### The enhanced activity of alternative tyrosine kinase signaling pathways by hTERT in the presence of PKC412

**Fig. 5:**
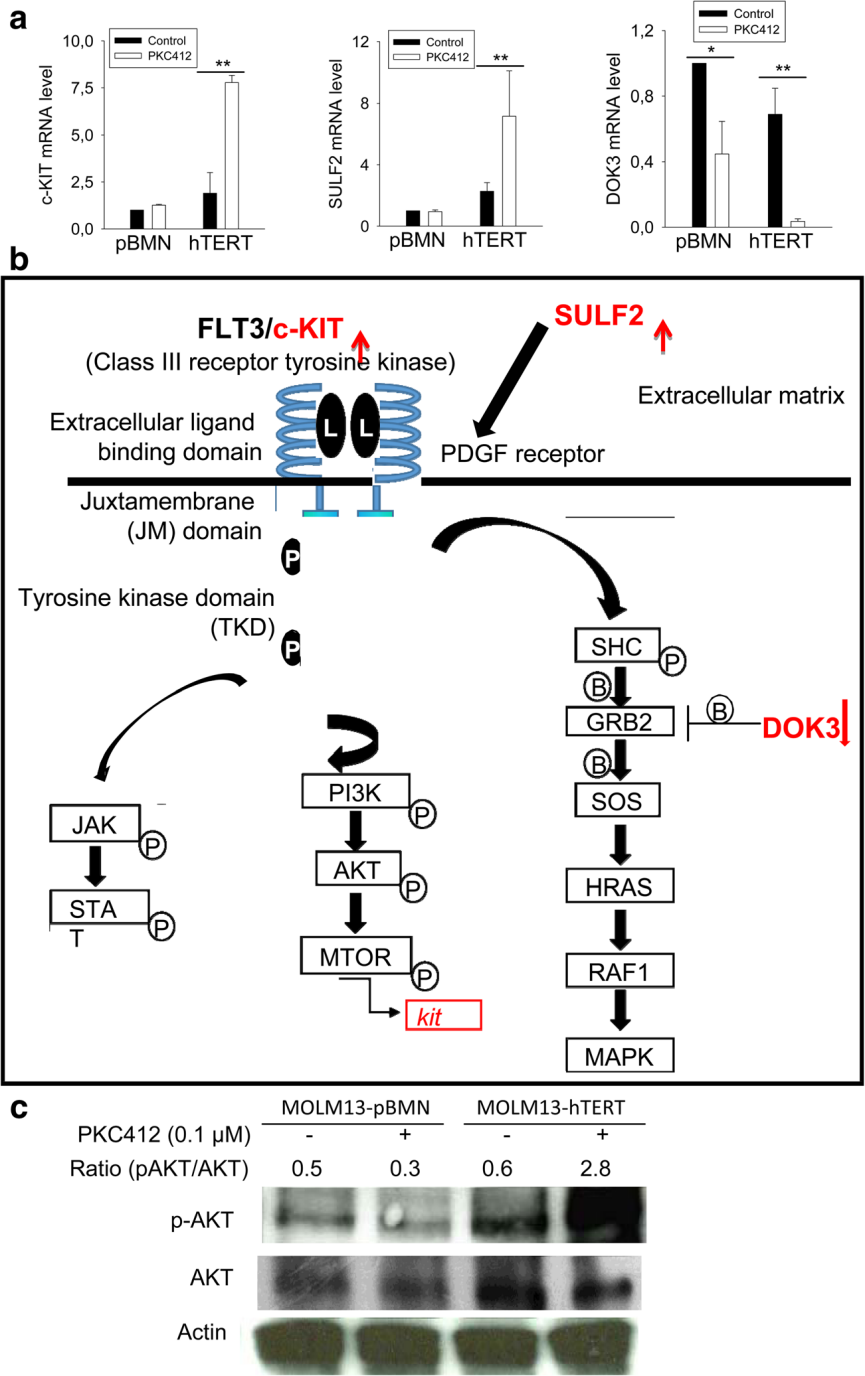
hTERT stimulation of the FLT3 downstream effectors and alternative tyrosine kinase (TK) pathways in the presence of PKC412. **a** Differential expression of c-KIT, DOC3, and SULF2 in MOLM-13-pBMN and MOLM-13-hTERT cells in the presence of PKC412. Cells were treated with PKC412 at 0.1 μM for 24 h, and the mRNA levels of three genes were determined using qPCR. *P < 0.05; ** *P* < 0.01. Three independent experiments were performed. **b** The schematic of c-KIT, DOC3, and SULF2 as regulators of the FLT3 and other RTK signaling pathways. **c** The enhanced Ser473 phosphorylation of AKT in PKC412-treated MOLM-13-hTERT cells. The signal ratios of pAKT/total AKT normalized to Actin were shown. Two independent experiments were performed with similar results

To probe how hTERT confers the cells resistance to PKC412-mediated apoptosis, we compared differences in gene expression profiles between MOLM-13-pBMN and MOLM-13-hTERT cells in the presence or absence of PKC412. Intriguingly, we identified that the ectopic hTERT expression significantly affected FLT3 and other RTK signaling pathways (Fig. 5a). First, the expression of c-KIT, another TK receptor structurally similar to FLT3, was upregulated by the ectopic hTERT expression, and its mRNA level was even much higher upon the exposure of MOLM-13-hTERT cells to PKC412 (Fig. 5a). In contrast, c-KIT expression did not change in MOLM-13-pBMN cells with and without PKC412. Second, DOC3, an endogenous inhibitor of the RSA-MAPK signaling, was downregulated in MOLM-13-hTERT cells and the PKC412 treatment led to further dramatic decline in DOC3 levels (Fig. 5a). Finally, SULF2 which activates the PDGF signaling pathway exhibited enhanced expression in MOLM-13-hTERT cells and its robust increase was observed following PKC412 treatment of these cells, whereas there was no detectable alteration in its expression in MOLM-13-pBMN cells with and without PKC412 (Fig. 5a, b).

### The enhanced AKT phosphorylation in MOLM-13-hTERT cells in the presence of PKC412

One of the key downstream effectors in TK signaling pathways is AKT, a pro-survival factor. Given all the above observations, we determined the AKT Ser473 phosphorylation between MOLM-13-pBMN and MOLM-13-hTERT cells with and without PKC412. As shown in Fig. 5c, the ectopic hTERT expression led to highly increased AKT phosphorylation in PKC412-treated cells.

## Discussion

The study presented here shows that FLT3ITD is required for constitutive hTERT transcription and sustained telomerase activity in MOLM-13 and MV4,11 cells. Inhibiting FLT3ITD by a specific TKI PKC412 led to diminished hTERT expression and telomerase activity. PKC412-mediated downregulation of hTERT is likely important for apoptotic cell death induced by FLT3ITD inhibition, because ectopic hTERT expression significantly increased cell survival of PKC412-treated MOLM-13 cells. Collectively, our findings reveal a functional link between the mutant FLT3 and hTERT or telomerase, which may be implicated in AML pathogenesis and therapy.

The induction of hTERT transcription and subsequent activation of telomerase is a critical step in malignant transformation. A key function of many oncogenic factors is to stimulate hTERT expression and telomerase activity. For instance, the pro-oncogene c-MYC is a master transcription factor activating *hTERT* gene transcription [27, 32]. Here, we demonstrated that FLT3ITD mediated MYC-dependent hTERT regulation. When FLT3ITD is inhibited by PKC412, rapidly diminished c-MYC expression occurred, and further analysis of the hTERT promoter showed that the MYC-binding motifs on the promoter were required for PKC412-mediated repression of the hTERT transcription. Of note, the PKC412 effect occurred only in FLT3ITD-carrying MOLM-13 and MV4–11 cell lines but not in HL60 or HeLa cells bearing wt FLT3. Although MOLM-13 and MV4–11 cell lines also harbor MLL mutations, there is no evidence that PKC412 interfere with the MLL pathway. Taken together, FLT3ITD upregulation of hTERT and telomerase activities contributes to the AML pathogenesis.

In addition to its telomere-lengthening function through which cellular telomere size is stabilized and malignant transformation thereby occurs, recent evidence has further demonstrated multiple biological activities of hTERT independent of a telomere elongation effect [16, 21–23, 25, 33–37]. These non-canonical functions of TERT facilitate proliferation of both normal and malignant cells and/or enhance their resistance to various stressful insults. Consistently, previous studies showed that high expression of hTERT and telomerase made cancer cells resistant to conventional chemotherapeutic drugs and radiotherapy as well. [17, 38] However, it is unclear whether this is the case in targeted cancer therapy against FLT3 mutation. To address this issue, we utilized the targeted therapeutic reagent PKC412, specifically to FLT3ITD, to treat FLT3ITD-carrying AML MOLM-13 cells. We found that the ectopic expression of hTERT significantly prevented apoptosis of MOLM-13 cells induced by PKC412, which suggests that hTERT does have a protective effect against targeted cancer therapy. On the other hand, PKC412 decreased S and G2/M cells independently of hTERT expression, and thus, hTERT does not affect PKC412-mediated cell cycle arrest.

Evidence has accumulated that the telomere-lengthening-independent function of hTERT or telomerase is achieved through its direct or indirect upregulation of growth factors and/or their receptors and pro-survival factors [21, 23, 33, 37]. By analyzing the expression profile in PKC412-treated cells, we found that hTERT significantly affected the FLT3 downstream effector PI3K-AKT, thereby exerting its action against the PKC412-induced apoptosis. The ectopic hTERT expression substantially enhanced the expression of c-KIT, another TK receptor structurally and functionally similar to FLT3, and c-KIT level was even much higher in the presence of PKC412. The increased c-KIT expression likely compensates for diminished AKT and MAPK activities due to FLT3ITD inhibition by PKC412. In addition, the ectopic hTERT expression in the presence of PKC412 upregulated the expression of SULF2, an activator in the PDGF signaling pathway. Intriguingly, DOC3, a negative regulator of the RAS signaling pathway, was highly repressed by hTERT over-expression, especially when cells were exposed to PKC412. Collectively, all these changes might result in partial recovery of the inhibited FLT3 downstream effectors in the presence of PKC412. Indeed, we observed the increased accumulation of phosphorylated AKT in MOLM-13-hTERT cells treated with PKC412. Because AKT is a well-established pro-survival factor, higher levels of phosphorylated AKT might protect cells against apoptosis, consistent with what we documented in PKC412-treated MOLM-13-hTERT cells. However, it remains unclear why the upregulation of SULF2, c-KIT expression, and AKT phosphorylation occur up on PKC412 exposure in hTERT-over-expressed cells, which calls for further investigations.

In summary, we identified that PKC412, a TKI specifically targeting FLT3ITD mutation, repressed hTERT transcription and telomerase activity in FLT3ITD-carrying AML cells in a MYC-dependent manner. This effect of PKC412 is likely associated with its therapeutic efficacy on AML. Importantly, we demonstrate that hTERT significantly attenuates the apoptotic cell death mediated by PKC412, which strongly indicates that hTERT is capable of mediating resistance to cancer-targeted therapy. Collectively, the present findings reveal a positive feedback link between FLT3ITD and telomerase and may have an important implication in AML pathogenesis and targeted therapy for both AML and other malignancies.
